# New Delhi Metallo-β-Lactamase–Producing *Enterobacterales* Bacteria, Switzerland, 2019–2020

**DOI:** 10.3201/eid2710.211265

**Published:** 2021-10

**Authors:** Jacqueline Findlay, Laurent Poirel, Julie Kessler, Andreas Kronenberg, Patrice Nordmann

**Affiliations:** University of Fribourg, Fribourg, Switzerland (J. Findlay, L. Poirel, J. Kessler, P. Nordmann);; University of Bern, Bern, Switzerland (A. Kronenberg);; Institute for Microbiology, University of Lausanne and University Hospital Centre, Lausanne, Switzerland (P. Nordmann);; Associate Editor, Emerging Infectious Diseases (P. Nordmann)

**Keywords:** *Enterobacterales*, *Klebsiella pneumoniae*, *Escherichia coli*, NDM, metallo-β-lactamase, New Delhi metallo-β-lactamase, Switzerland, carbapenemase, bacteria, antimicrobial resistance

## Abstract

Carbapenemase-producing *Enterobacterales* (CPE) bacteria are a critical global health concern; New Delhi metallo-β-lactamase (NDM) enzymes account for >25% of all CPE found in Switzerland. We characterized NDM-positive CPE submitted to the Swiss National Reference Center for Emerging Antibiotic Resistance during a 2-year period (January 2019–December 2020) phenotypically and by using whole-genome sequencing. Most isolates were either *Klebsiella pneumoniae* (59/141) or *Escherichia coli* (52/141), and >50% were obtained from screening swabs. Among the 108 sequenced isolates, NDM-1 was the most prevalent variant, occurring in 56 isolates, mostly *K. pneumoniae* (34/56); the next most prevalent was NDM-5, which occurred in 49 isolates, mostly *E. coli* (40/49). Fourteen isolates coproduced a second carbapenemase, predominantly an OXA-48-like enzyme, and almost one third of isolates produced a 16S rRNA methylase conferring panresistance to aminoglycosides. We identified successful plasmids and global lineages as major factors contributing to the increasing prevalence of NDMs in Switzerland.

Carbapenem-resistant *Enterobacterales* (CRE) bacteria are considered by the World Health Organization to be a critical global health concern; they were placed in the organization’s critical-priority group of the priority pathogens list for the research and development of new antibiotics in 2017 (*1*). Among the Ambler class B β-lactamases, the New Delhi metallo-β-lactamases (NDM) were identified in 2008 in a patient from Sweden who had been hospitalized in India and upon return to Sweden had a carbapenem-resistant *Klebsiella pneumoniae* sequence type (ST) 14 strain isolated from his urine, leading to the identification of the *bla*_NDM-1_ gene (*2*). In a follow-up study in 2009, NDM enzymes were shown to be widespread in India, Pakistan, and Bangladesh; the *bla*_NDM-1_ gene was identified in multiple *Enterobacterales* species, predominantly in *Escherichia coli* and *K. pneumoniae* (*3*). Since those initial studies, NDM carbapenemases have been reported globally (*4*,*5*). The SMART global surveillance program analyzed *Enterobacterale* isolates in 55 countries from 2008–2014 and found that the prevalence of NDM carbapenemase producers was substantially higher in India, Egypt, the United Arab Emirates, and Serbia (*6*). In 2010, NDM-1-producing *Acinetobacter baumannii* bacteria were reported in India (*7*), and reports in other *Acinetobacter* spp. followed (*8*). In 2011, NDM-1 was reported in *Pseudomonas aeruginosa* in Serbia (*9*), illustrating a wide host range among gram-negative bacteria.

NDM enzymes are capable of conferring resistance to almost all β-lactam antimicrobial drugs (except aztreonam), including carbapenems which are often considered drugs of last resort for the treatment of serious infections (*2*). Treatment options for infections caused by NDM-producing bacteria are very limited, particularly because they often harbor multiple other resistance genes. For example, there are notable associations between *bla*_NDM_ genes and plasmid-borne extended-spectrum β-lactamases (ESBLs) and pAmpC encoding genes (most commonly *bla*_CTX-M_ and *bla*_CMY_) that result in resistance to aztreonam (*10*); similarly, 16S rRNA methylases (RMTases), which can confer high-level resistance to all aminoglycosides, have also been strongly associated with NDM carriage (*11*). The recently approved β-lactamase inhibitors, diazabicyclooctanes (e.g., avibactam [AVI], relebactam [REL]) and cyclic boronates (e.g., vaborbactam [VAB]) have no activity against metallo-β-lactamases (MBLs) and subsequently new treatment options are urgently needed. Aztreonam (ATM)/AVI has been suggested as a treatment option for infections caused by NDM-producing bacteria because ATM is spared by MBL hydrolytic activities and AVI inhibits the activity of any co-produced ESBL or AmpC (*12*).

To date, a total of 32 NDM variants have been identified (https://www.ncbi.nlm.nih.gov/pathogens/beta-lactamase-data-resources); however, the NDM-1, NDM-4, NDM-5, and NDM-7 variants remain dominant globally with some exhibiting increased carbapenemase activity compared with NDM-1 (*3*–*5*,*10*). NDM encoding genes are highly transmissible, often located on plasmids of various replicon types harboring several antibiotic resistance genes. Outbreaks of NDM producers, either clonal or due to the dissemination of successful plasmids, have been increasingly reported (*13*–*15*).

In Switzerland, production of NDM enzymes was identified in 2011 in *E. coli*, *K. pneumoniae,* and *Proteus mirabilis* isolates obtained from Geneva University Hospitals (*16*) and since then have become one of the dominant carbapenemase types, just after oxacillin (OXA) 48 (*17*) in the country, accounting for >25% of all carbapenem-producing *Enterobacterales* (CPE) submitted to the Swiss National Reference Centre for Emerging Antibiotic Resistance (NARA) in 2020. In this study we describe the epidemiology of NDM-positive *Enterobacterales* submitted to NARA during January 2019–December 2020.

## Materials and Methods

### Bacterial Isolates, Identification, and Susceptibility Testing

The NARA reference laboratory received 532 CPE samples from hospitals and clinics throughout Switzerland over a 2-year period, January 2019–December 2020, after a mandatory request in January 2019 for carbapenemase producers. We obtained patient and isolation source data from the accompanying request forms sent by referring laboratories. Of the 532 samples, 141 were confirmed to be NDM-positive enterobacterial isolates. Species identification was confirmed using API-20E tests (bioMérieux, https://www.biomerieux.com) and UriSelect 4 agar (Bio-Rad, https://www.bio-rad.com). Susceptibility testing was performed by disk diffusion or by broth microdilution for the β-lactam/β-lactamase inhibitor combinations and results interpreted in accordance with EUCAST guidelines (*18*). For the ATM/AVI combination, AVI was used at a fixed concentration of 4 mg/L.

### Detection of Carbapenemase Genes

All isolates were subject to the Rapidec Carba NP test (bioMérieux) and then to NG-Test CARBA 5 test (NG Biotech, https://ngbiotech.com), according to the manufacturer’s instructions. Isolates testing positive by the Rapidec Carba NP test and the NG-Test CARBA 5 test or exhibiting resistance to >1 carbapenem (ertapenem, imipenem, or meropenem) were screened by PCR (*19*) for the presence of carbapenemase genes (*bla*_KPC_, *bla*_OXA-48_, *bla*_NDM_, *bla*_VIM_, and *bla*_IMP_). Sanger sequencing of amplified carbapenemase genes was performed by Microsynth AG (Microsynth AG, https://www.microsynth.com) to identify the exact alleles.

### Whole-Genome Sequencing and Analyses

Whole-genome sequencing (WGS) was performed on a subset of 108 nonduplicate isolates (by patient and species) on a MiSeq instrument (Illumina, https://www.illumina.com) using the Nextera sample preparation method with 2 × 150 bp paired end reads. Reads were assembled into contigs using the Shovill pipeline (https://github.com/tseemann/shovill), which is based on SPAdes version 3.13.0 (*20*). Sequence types, the presence of resistance genes, and speciation were confirmed, using MLST version 2.0, ResFinder version 4.1 (*21*), and KmerFinder version 3.2 (*22*) on the Center for Genomic Epidemiology platform (https://cge. cbs.dtu.dk/services); contigs were annotated using Prokka (*23*). A core genome single-nucleotide polymorphism (SNP) alignment was generated using Parsnp (*24*) and viewed using Interactive Tree of Life version 6.1.1 (*25*). *E. coli* MG1655 (GenBank accession no. NC_000913) and *K. pneumoniae* ATCC 700721/MGH78578 (GenBank accession no. NC_009648) were used as the reference sequences for each alignment.

Complete NDM-encoding plasmid sequences were downloaded from GenBank by using the search terms and filters “NDM” and “plasmid” and applying a minimum sequence length of 15 kb to generate an NDM plasmid reference database for mapping analyses. Reads were mapped to reference sequences using CLC Genomics Workbench (QIAGEN, https://www.qiagen.com) and then contigs mapped using progressive Mauve alignment software to manually mitigate against false positives (*26*). A >95% coverage and identity were used to assess relevant matches (Appendix Table). We have submitted sequence data from this study to the National Center for Biotechnology Information’s Sequence Read Archive (BioProject no. PRJNA744003). 

## Results

### Isolate Sources and Species Identification

More than half of isolates (82/141; 58.2%) were obtained from screening swab samples (fecal and rectal, and nonrectal) and the remaining isolates were from urine (34/141; 24.1%), tissue and fluid (14/141; 9.9%), respiratory (4/141; 2.8%), blood cultures (3/141; 2.1%), and 4 isolates were of unknown origin (Table 1). Isolates were *K. pneumoniae* (59/141; 41.8%), *E. coli* (52/141; 36.9%), *Enterobacter cloacae* complex (19/141; 13.5%), *Citrobacter freundii* (3/141; 2.1%), *Klebsiella aerogenes* (3/141; 2.1%), *Klebsiella quasipneumoniae* (2/141; 1.4%), *Klebsiella variicola* (2/141; 1.4%), and *Klebsiella oxytoca* (1/141; 0.7%). Isolates were obtained from 116 patients; 65 were male (56%), 47 female (41%), and 4 did not have sex stated.

**Table 1:** Sources of 141 *bla*_NDM_-positive isolates identified in *Enterobacterales* bacteria, Switzerland, 2019–2020

Species	No. from patient source							Total
	Urine	Blood culture	Tissue and fluid	Respiratory	Fecal and rectal swab	Nonrectal screening swab	Unknown	
*Klebsiella pneumoniae*	12	1	7	2	31	4	2	59
*Escherichia coli*	13	2	1	0	31	4	1	52
*Enterobacter cloacae* complex	6	0	5	1	6	0	1	19
*K. aerogenes*	1	0	0	0	2	0	0	3
*Citrobacter freundii*	1	0	0	0	1	1	0	3
*K. quasipneumoniae*	0	0	0	0	2	0	0	2
*K. variicola*	0	0	1	1	0	0	0	2
*K. oxytoca*	1	0	0	0	0	0	0	1
Total	34	3	14	4	73	9	4	141

**Figure 1:**
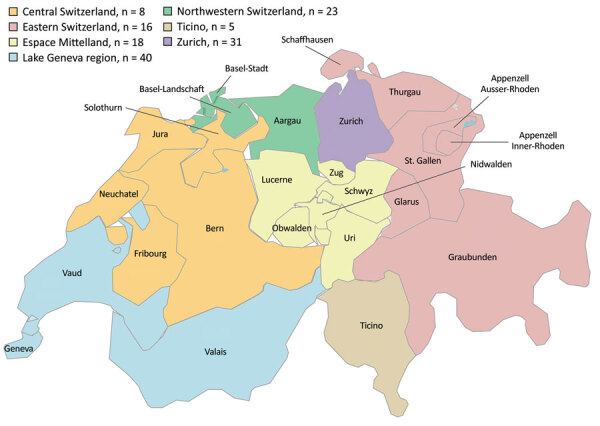
Regions of Switzerland from which *Enterobacterale* isolates positive for New Delhi metallo-β-lactamase were obtained, January 2019–December 2020.

All 7 main regions of Switzerland were represented in this study; 8–40 isolates were submitted from each (Figure 1). Approximately half of all isolates (71/141; 50.4%) were obtained from hospitals in either the Lake Geneva or Zurich region, 2 of the most populated areas of Switzerland, but just 8 isolates were received from central Switzerland, the region with the greatest population size (*27*). Sixty-six isolates were from 2019 and 75 from 2020, whereas 33 NDM-positive *Enterobacterales* isolates were submitted to NARA in 2018 (data not shown). Such a significant increase in numbers could indicate a trend of increased NDM prevalence in Switzerland, as has been observed previously during 2013–2018 (*17*). We selected 108 nonduplicate isolates for further investigation: 46 *E. coli*, 42 *K. pneumoniae*, 14 *Enterobacter cloacae* complex, 3 *K. quasipneumoniae*, 2 *K. aerogenes*, and 1 *K. pneumoniae variicola* isolate.

### Susceptibility Testing

Susceptibility testing showed that most isolates were resistant to fluoroquinolones (93/108; 86.1%), and most (69/108; 63.9%) were resistant to >2 aminoglycosides, of which we tested kanamycin, tobramycin, gentamicin, and amikacin. No isolate was found resistant to tigecycline, 6 (5.6%) isolates exhibited resistance to colistin, and 1 isolate was resistant to fosfomycin. We also performed susceptibility testing against selected β-lactam and β-lactam/inhibitor combinations, namely imipenem (IPM), IPM/REL, meropenem (MEM), MEM/VAB, ceftazidime (CAZ), CAZ/AVI, ATM, and ATM/AVI (Table 2). All isolates were resistant to both CAZ and CAZ/AVI, as well as MEM; 17 (15.7%) isolates were susceptible to MEM/VAB. Ten (9.3%) isolates were susceptible to IPM and 2 (1.9%) to IPM/REL; of note, breakpoints for IPM and IPM/REL are different, which may explain the lower susceptibility rate for the combination. Most (97/108; 89.8%) isolates exhibited resistance to ATM, whereas 8 isolates (7 *E. coli* and 1 *K. pneumoniae*) were resistant to ATM/AVI with MICs of 8 mg/L (n = 4) and 16 mg/L (n = 4), based on breakpoint value of resistance for AZT/AVI taken from that of ATM. Among the drug combinations, AZT/AVI was the most effective, as expected.

**Table 2:** MIC distributions for 108 *bla*_NDM_-positive isolates identified in *Enterobacterales* bacteria, Switzerland, 2019–2020*

Antimicrobial drug	Breakpoint MIC, mg/L			No. isolates at MIC													% Susceptible
	S	R		<0.06	0.125	0.25	0.5	1	2	4	8	16	32	64	128	>256	
Imipenem	<2	>4							2	8	23	37	19	11	8		9.3
Imipenem/relebactam	<2	>2							2	9	30	34	15	11	7		1.9
Meropenem	<2	>2								8	9	15	27	25	20	4	0
Meropenem/vaborbactam	<2	>8								8	9	19	27	25	19	1	15.7
Ceftazidime	<1	>4														108†	0
Ceftazidime/avibactam	<8	>8														108†	0
Aztreonam	<1	>4			3	1	1	1	2	3	8	3	2	8	22	54	10.2
Aztreonam/avibactam‡	≤4	>4§		15¶	19	16	14	12	11	13	4	4					92.6

*Testing range for all drugs was 0.06–256 mg/L. Breakpoints from the European Committee on Antimicrobial Susceptibility Testing. Gray shading indicates resistant isolates. R, resistant; S, susceptible. †MIC>256 mg/L. ‡Avibactam used at a fixed concentration of 4 mg/L. §Aztreonam/avibactam breakpoint selected arbitrarily based on the aztreonam breakpoints. ¶MIC<0.06 mg/L.

#### AZT/AVI Resistance Mechanisms

Analysis of the ATM/AVI-resistant isolates revealed that 6/7 *E. coli* isolates harbored a *bla*_CMY_ allele: *bla*_CMY-2_ (n = 1), *bla*_CMY-42_ (n = 2), *bla*_CMY-145_ (n = 2), and *bla*_CMY-148_ (n = 1). All those isolates had an insertion of 4 amino acids within the PBP-3 encoding gene located after residue 333, which was YRIN in 5 isolates and YRIK in the other 2 isolates. Similar 4-aa insertions have been reported among NDM-5–producing *E. coli* as a cause of elevated MICs of ATM, related to impeding accessibility to the binding site of PBP-3, and therefore were involved in ATM/AVI resistance in addition to CMY production (*28*). Ma et al. reported that the insertion alone has a minor effect on ATM/AVI resistance levels (*29*), but resistance could be achieved when combined with CMY production (*29*). Other studies reported that CMY variants with a glycine residue at position 231 exhibited enhanced hydrolysis against ATM (*30*,*31*). Among the isolates we tested, CMY-42, CMY-145, and CMY-148 variants all harbored a Val231Ser substitution, suggesting that those enzymes affected the levels of ATM/AVI resistance, along with the PBP-3 modifications.

One ATM/AVI-resistant *K. pneumoniae* isolate exhibited an MIC of 8 mg/L; it neither carried a *bla*_CMY_ gene nor harbored any mutation within its PBP-3 encoding gene. However, it did harbor *bla*_CTX-M-15_, and we identified a premature stop codon near the beginning of *ompK35*, which would render the porin nonfunctional. Although this does not explain the ATM/AVI MIC by itself, it might be a contributing factor.

### Carbapenemase Alleles and Other Resistance Genes

#### Carbapenemase Alleles

**Figure 2:**
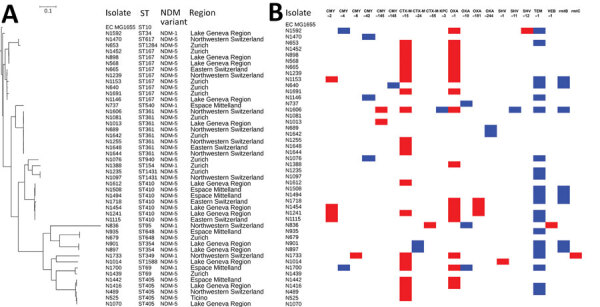
Clustering and gene content of 46 *Escherichia coli* isolates collected in Switzerland, January 2019–December 2020. A) Phylogenetic tree showing clustering and the respective ST, NDM variant, and region of Switzerland from which each isolate was obtained. B) Gene matrix showing β-lactamase and RMTase gene content of the isolates. NDM, New Delhi metallo-β-lactamase; ST, sequence type.

**Figure 3:**
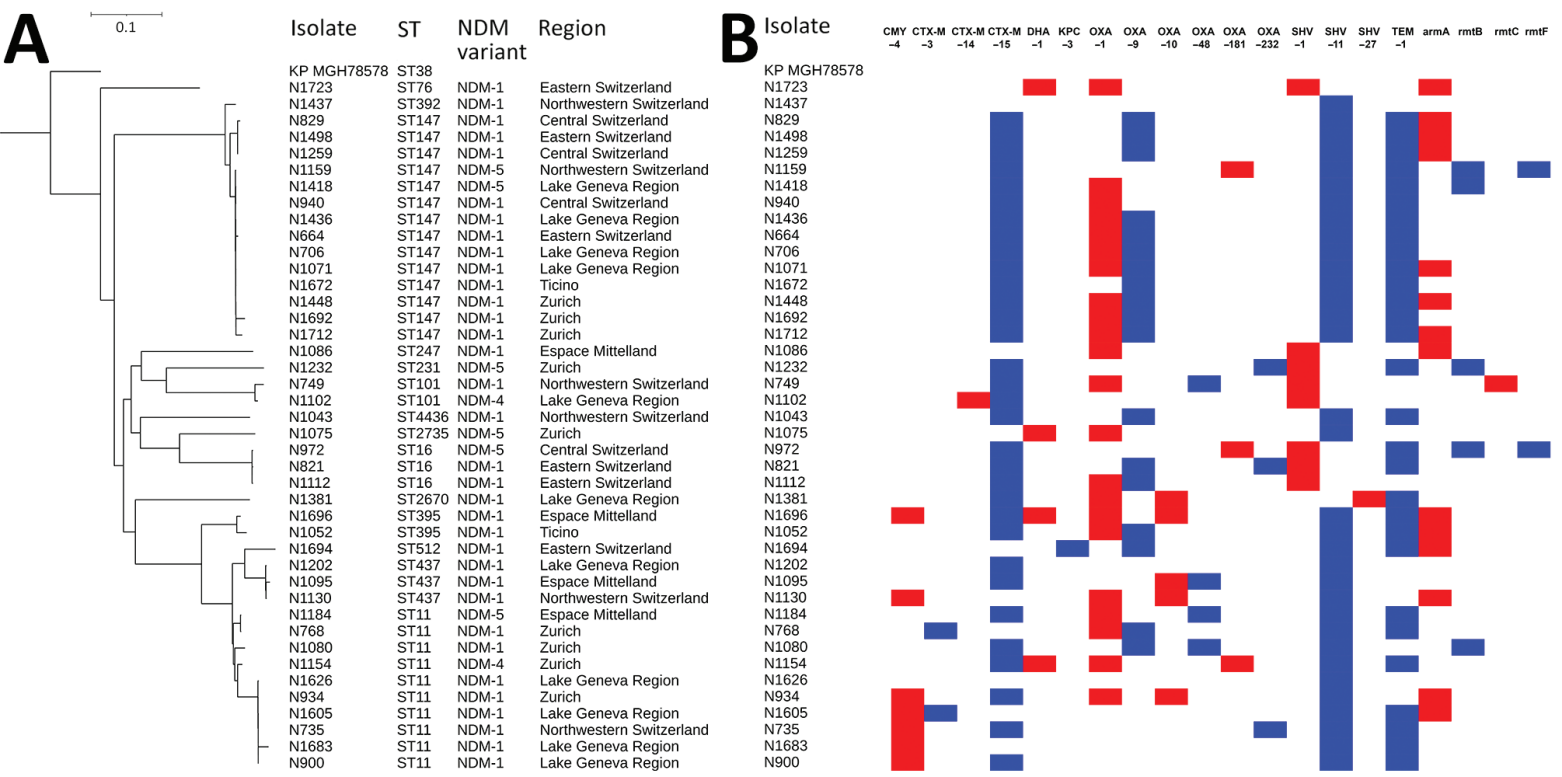
Clustering and gene content of 42 *Klebsiella pneumoniae* isolates collected in Switzerland, January 2019–December 2020. A) Phylogenetic tree showing clustering and the respective ST, NDM variant, and region of Switzerland from which each isolate was obtained. B) Gene matrix showing β-lactamase and RMTase gene content of the isolates. NDM, New Delhi metallo-β-lactamase; ST, sequence type.

Among the 108 sequenced isolates, we identified 4 *bla*_NDM_ allelic variants; *bla*_NDM-1_ (n = 56), *bla*_NDM-4_ (n = 2), *bla*_NDM-5_ (n = 49), and *bla*_NDM-7_ (n = 1). Most *E. coli* isolates harbored *bla*_NDM-5_ (40/46 isolates) and the 6 remaining *E. coli* isolates had *bla*_NDM-1_ (Table 3). This finding indicated that the spread of *bla*_NDM-5_ gene in *E. coli* may be affected by the increased catalytic efficiency of NDM-5 against carbapenems compared with NDM-1 (*32*). Conversely, *K. pneumoniae* isolates predominantly carried *bla*_NDM-1_ (34/42), and rarely *bla*_NDM-5_ (6/42) and *bla*_NDM-4_ (2/42). Most (12/14) *E. cloacae* complex isolates harbored *bla*_NDM-1_; of the others, 1 harbored *bla*_NDM-5_ and 1 *bla*_NDM-7_. Multiple carbapenemase genes were found in 18 isolates, namely 11 *K. pneumoniae*, 6 *E. coli,* and 1 *E. cloacae* complex isolate. Sixteen of the 18 isolates harbored a *bla*_OXA-48_-like gene (*bla*_OXA-48_ [n = 5], *bla*_OXA-181_ [n = 6], *bla*_OXA-232_ [n = 3], *bla*_OXA-244_ [n = 2]), and single *E. coli* and *K. pneumoniae* isolates harbored *bla*_KPC-3_ genes. In addition to producing the various carbapenemases, most isolates also produced multiple other β-lactamases, including CTX-M–type ESBLs and CMY-type AmpC–encoding genes. We identified genes encoding RMTases conferring resistance to all clinically significant aminoglycosides in a total of 35 isolates (Figures 2, 3).

**Table 3:** Carbapenemase alleles harbored by isolates sequenced in study of *Enterobacterales* bacteria, Switzerland, 2019–2020

Species	Carbapenemase													
	NDM-1	NDM-1 + OXA-48	NDM-1 + OXA-232	NDM-1 + KPC-3	NDM-4	NDM-4 + OXA-181	NDM-5	NDM-5 + OXA-48	NDM-5 + OXA-181	NDM-5 + OXA-232	NDM-1 + OXA-244	NDM-5 + KPC-3	NDM-7	Total
*Escherichia coli*	6						34		3		2	1		46
*Klebsiella pneumoniae*	28	3	2	1	1	1	2	1	2	1				42
*Enterobacter cloacae* complex	11	1					1						1	14
*K. aerogenes*							2							2
*K. quasipneumoniae*	3													3
*K. variicola*	1													1
Total	49	4	2	1	1	1	39	1	5	1	2	1	1	108

#### *E. coli* Isolates

We identified a total of 17 sequence types (STs) among the 46 *E. coli* isolates. Four dominant ST clusters or clonal complexes (CCs) were identified by performing core genome SNP analyses as follows: ST405 (n = 5), all producing NDM-5 and obtained from 4 regions of Switzerland; ST410 (n = 7), all producing NDM-5 and obtained from 3 regions; ST361 (n = 8), all producing NDM-5 and obtained from 4 regions; CC10 from 4 regions, comprising ST167 (n = 9) and single representatives of its single locus variants, ST617 and ST1284, all of which also produced NDM-5 (Figure 2). Six isolates co-produced another carbapenemase gene, namely *bla*_OXA-181_ (n = 3), *bla*_OXA-244_ (n = 2), or *bla*_KPC-3_ (n = 1). The core genome size in this analysis was 2.82 Mb.

#### *K. pneumoniae* Isolates

Within the 42 *K. pneumoniae* isolates, we identified 14 STs. A core-genome SNP analysis showed that 2 clonal clusters dominated; 1 contained CC258 isolates, comprising 10 ST11 and 3 ST437 isolates, and the other included 14 ST147 isolates (Figure 3). Among CC258 isolates, all produced NDM-1 with the exception of 1 that produced NDM-4 and 1 NDM-7, both belonging to ST11. Within ST147 isolates, 12 produced NDM-1 and 2 produced NDM-5. Isolates from both clusters were scattered geographically; we obtained CC258 isolates from hospitals in 4 regions and ST147 isolates from 6 regions in Switzerland. Eleven isolates coharbored >1 carbapenemase gene; the genes were *bla*_OXA-48_ (n = 4), *bla*_OXA-181_ (n = 3), *bla*_OXA-232_ (n = 3), and *bla*_KPC-3_ (n = 1). The core genome size in this analysis was 4.25 Mb.

#### *E. cloacae* Complex Isolates and Other Species

The 14 *E. cloacae* complex isolates represented 10 STs, indicating no dominant clone. Twelve isolates produced NDM-1, and the remaining 2 produced either NDM-5 or NDM-7. One ST91 *E. cloacae* isolate additionally carried a *bla*_OXA-48_ gene. The remaining isolates included 3 of *K. quasipneumoniae*, 2 of *K. aerogenes*, and 1 of *K. variicola.* The *K. quasipneumoniae* isolates were of ST4834, ST5330, and 1 novel ST. Both *K. aerogenes* isolates belonged to ST93. The *K. quasipneumoniae* and *K. variicola* isolates produced NDM-1, and the *K. aerogenes* isolates produced NDM-5.

#### 16S RMTases

By screening our collection of NDM-producing isolates for RMTase encoding genes, we found a high positivity rate. Almost a third of isolates (35/108) harbored >1 RMTase gene, most commonly *armA* (16 isolates), followed by *rmtB* (13 isolates), *rmtC* (6 isolates), and *rmtF* (co-produced in 2 isolates alongside *rmtB*). RMTases are capable of conferring high-level resistance to all clinically relevant aminoglycoside antimicrobial drugs including the recently approved urinary tract infection treatment plazomicin (*33*). RMTases have previously been shown to have a strong association with *bla*_NDM_, particularly *K. pneumoniae* ST147 (*34*); in our study, 8 of the *K. pneumoniae* ST147 isolates carried RMTase genes. Of the 35 RMTase-positive isolates we identified, a highly similar plasmid could be identified for 24, and 10/24 harbored the same RMTase genes as the reference plasmid. This result could suggest that the RMTase genes in these isolates may be carried on the same plasmid as the *bla*_NDM_ gene. The remaining 14 identified highly similar plasmids either did not encode an RMTase gene or encoded one different from that identified in our isolates. The strong association between *bla*_NDM_ and RMTase gene carriage have been previously reported elsewhere (*34*) and has been associated with both the co-localization of *bla*_NDM_ and RMTase gene types on the same plasmid, as well as encoded separately in diverse plasmid types (*34*). Such high level of association of carbapenemases to the NDM-5 and RMTase genes will further limit the choice of therapeutics available for treating infections because of those multidrug-resistant bacteria.

### Typing of NDM Plasmids

By mapping sequencing reads against a database of known NDM-encoding plasmids and applying a stringent cutoff (>95% coverage and identity), we identified plasmids highly similar to those in our study. We found plasmids with >95% coverage and identity for 69/108 (63.9%) of the sequenced isolates (Appendix Table). Most (50/69) of the identified plasmids harbored IncF replicons, albeit a diverse range. Plasmids with the IncF replicon types were the most common, among which the *bla*_NDM-5_ gene was dominant; the replicons IncFII (n = 14), IncFII/IncFIA (n = 11), IncFIB(pQIL) (n = 7), and IncF(pKPX1) (n = 6) were the most common. A total of 13/69 plasmids carrying a *bla*_NDM_ gene possessed an IncX3 replicon, and carried either *bla*_NDM-5_ (n = 9), *bla*_NDM-1_ (n = 3), and *bla*_NDM-7_ (n = 1) genes.

Within *E. coli* isolates, highly similar plasmids could be identified for 31/46 isolates, representing 7 different replicon types and combinations. Most (24/31) were IncF replicon variants, and 9 of these *bla*_NDM_-bearing plasmids exhibited 95%–100% coverage and identity to pIncF, a ≈116 kb IncFII/IncFIA NDM-5–producing plasmid identified in *E. coli* ST617 from Italy (GenBank accession no. MW048884.1). The pIncF-like plasmids were identified in 4 STs, namely ST167 (n = 4), ST361 (n = 3), ST617 (n = 1), and ST1588 (n = 1). Highly similar IncX3 plasmids could be identified from 4 isolates, corresponding to the previously reported ≈46 kb pEsco-5256cz (GenBank accession no. MG252891.1) carrying the *bla*_NDM-5_ gene from Czech Republic, and 2 other highly similar *bla*_NDM-5_-carrying IncX3 plasmids exhibited high similarity to a ≈35 kb pABC280-NDM-5 (GenBank accession no. MK372392) identified from the United Arab Emirates.

Within *K. pneumoniae* isolates, we identified similar plasmids for 28/42 isolates from 9 different replicon types or combinations. Similar to *E. coli* isolates, most plasmids (22/28) corresponded to IncF replicon variants. Seven isolates, all belonging to ST147, exhibited 99%–100% coverage and identity to pM321-NDM-1 (GenBank accession no. AP018834), a ≈54 kb *bla*_NDM-1_-positive plasmid harboring the FIB(pQIL) replicon type and described in isolates from Myanmar (*35*). Six ST11 *K. pneumoniae* isolates also exhibited 100% coverage and similarity to pAR_0146 (GenBank accession no. CP021962), a ≈ 132 kb IncFII(pKPX1) plasmid identified in the United States. Of interest, within the 4 IncX3 plasmids that could be identified, 2 exhibited high similarity (100% coverage and identity) to pEsco-5256cz and 1 to pABC280-NDM-5; we found those 2 plasmids in *E. coli* isolates as well, which suggested interspecies plasmid sharing. We also detected the plasmid pEsco-5256cz in the *E. cloacae* complex isolates; 3 of those ST93 isolates harboring plasmids exhibited 99% coverage and identity to pEsco-5256cz. 

## Discussion

This study showed increasing prevalence of NDM-producing *Enterobacterales* bacteria in Switzerland. One cause appears to be successful lineages of both *E. coli* and *K. pneumoniae*. 

The *E. coli* clusters we identified in this study are all considered as high-risk clones or correspond to CC that are frequently reported internationally (*36*–*40*). Multiple studies reported NDM-5–producing ST405 *E. coli* isolates circulating in Europe and particularly in isolates from Germany, Italy, and Spain (*39*–*40*). One study alluded to the cross-border transmission between Switzerland and Germany of NDM-5-producing ST405 isolates (*39*), and in our study this *E. coli* clone also appears widespread, found in multiple regions of Switzerland. *E. coli* ST410 is increasingly reported as a cause of hospital outbreaks and has been associated with the carriage of both ESBLs and various carbapenemase genes, including *bla*_NDM-5_, in both Europe and Asia (*36*,*37*). CC10 members producing NDM-5, have been described in multiple countries, including China, the United States, and Switzerland (*39*,*41*). We found it in isolates across a wide geographical area. *E. coli* ST361 was most commonly described as an ESBL-producing clone in both human and animal populations, usually harboring the *bla*_CTX-M-15_ gene, but more recently it has been described as harboring *bla*_NDM-5_ in several countries, including Germany and Switzerland (*39*,*42*–*45*). Similarly, ESBL-producing *E. coli* ST167 and ST410 isolates have been found in food products (meat and dairy) in Germany (*45*), indicating that these lineages may already be widespread, albeit without the *bla*_NDM-5_–carrying plasmid. A recent study in Switzerland identified both ST167 and ST410 *E. coli* isolates harboring the NDM-5 encoding gene; genomic analysis showed that the isolates harbored *bla*_NDM-5_–carrying plasmids with a high nucleotide identity to plasmids previously identified in a nosocomial outbreak in Myanmar (*38*).

Both *K. pneumoniae* clusters identified in this study encompass high-risk clones. *K. pneumoniae* ST147 has emerged as an important clone for the dissemination of various antimicrobial-resistance genes, given its wide global distribution and strong association with hospital outbreaks (*46*). Tavoschi et al. identified NDM-1-producing ST147 *K. pneumoniae* isolates as the dominant cause of a yearlong outbreak in hospitals in Tuscany, Italy (*42*); their finding might explain a dominance of ST147 isolates, considering the proximity of Italy and Switzerland. *K. pneumoniae* CC258 is most commonly associated with the global dissemination of *bla*_KPC_ through ST11 and ST258. *K. pneumoniae* ST11, however, seems amenable to the dissemination of a wide range of resistance genes; hospitals have reported outbreaks harboring various carbapenemase family genes including *bla*_KPC_, *bla*_NDM_, and *bla*_VIM_ (*47*,*48*). NDM-1–producing *K. pneumoniae* ST11 has long been reported throughout Europe and could be considered as endemic in some countries (*48*–*50*). *K. pneumoniae* ST11 might therefore be considered as a successful clone in Switzerland, as we observed. We detected pEsco-5256cz–like and pABC280-NDM-5–like IncX3 plasmids in complex isolates of all 3 species groups (*E. coli*, *K. pneumoniae,* and *E. cloacae*). Our findings suggest that the plasmids are highly capable of cross-species transmission, which has been observed for IncX3 plasmids generally and is a factor in their success.

The isolates in this study were multidrug resistant, and a substantial proportion exhibited resistance to aminoglycosides, largely resulting from the co-carriage of RMTases. The high level of association of carbapenemases to the NDM-5 and RMTase genes will limit the choice of therapeutics available for treating infections because of those multidrug-resistant bacteria. Several isolates were identified that were resistant to the β-lactam/inhibitor combination ATM/AVI, a potential future treatment for infections caused by NDM-producing bacteria (*28*,*29*). The transmission of successful plasmids, both within and between species, was identified as a major factor in the increasing prevalence of NDM-producing *Enterobacterales*. This 2-year study gives a snapshot of the epidemiology of NDM producers in Switzerland and illustrates how the use of WGS is both an essential and informative tool for surveillance and for monitoring emerging resistance. Our findings underpin the importance of the surveillance of NDM-producing bacteria and particularly the monitoring of successful clonal lineages and plasmids.

Appendix Additional information about New Delhi metallo-β-lactamase–producing *Enterobacterales* bacteria in Switzerland, 2019–2020.
