# Supplementary material for: New Delhi Metallo-β-Lactamase–Producing Enterobacterales Bacteria, Switzerland, 2019–2020
Source: Emerg Infect Dis. 2021 Oct;27(10):2628–37. doi: 10.3201/eid2710.211265 (PMC8462332; doi:10.3201/eid2710.211265)
Supplement: Appendix — Additional information about New Delhi metallo-β-lactamase–producing Enterobacterales bacteria in Switzerland, 2019–2020. [file 21-1265-Techapp-s1.pdf]

Article DOI: https://doi.org/10.3201/eid2710.211265

New Delhi Metallo-β-Lactamase–Producing *Enterobacterales* Bacteria, Switzerland, 2019–2020

Appendix

Appendix Table. Plasmid mapping analyses performed on all 108 sequenced isolates in study of *Enterobacterales* bacteria, detailing highly similar plasmids (>95% coverage and identity), replicon types, and RMTase genes identified in both reference plasmids and the study isolates.

Isolate	Species	ST	NDM variant	Plasmid mapping/ID (%)	GenBank accession no.	Plasmid size, bp	Country in which described	Replicon	RMTase ID'd in ref plasmid	RMTase ID'd in isolate
N489	*Escherichia coli*	405	NDM-5	pYJ4-NDM-5, 100)	AP023231	83453	Myanmar	FII		
N525	*Escherichia coli*	405	NDM-5	pCH18NDM5 (100)	PRJNA630933	86167	Switzerland	FII		
N568	*Escherichia coli*	167	NDM-5	ND	ND	ND	ND	ND		
N640	*Escherichia coli*	167	NDM-5	pM217-FII (100)	AP018147	102071	Myanmar	FII	rmtB	rmtB
N653	*Escherichia coli*	1284	NDM-5	pNDM-5-1001 (95)	MH985167	90378	China	FII, FIA, FIB(AP001918)		
N665	*Escherichia coli*	167	NDM-5	pEsco-5256cz (100)	MG252891	46161	Czechia	X3		
N679	*Escherichia coli*	648	NDM-5	pCH11NDM5 (100)	PRJNA630933	124740	Switzerland	FII, FIBpB171		
N689	*Escherichia coli*	361	NDM-5	ND	ND	ND	ND	ND		
N737	*Escherichia coli*	540	NDM-1	ND	ND	ND	ND	ND		
N836	*Escherichia coli*	95	NDM-1	ND	ND	ND	ND	ND		
N897	*Escherichia coli*	354	NDM-5	p1ESCUMpO83 (95)	CP034254	92840	India	FII	rmtB	rmtB
N898	*Escherichia coli*	167	NDM-5	ND	ND	ND	ND	ND		
N901	*Escherichia coli*	354	NDM-5	p1ESCUMpO83 (95)	CP034254	92840	India	FII	rmtB	rmtB
N935	*Escherichia coli*	648	NDM-5	pCH11NDM5 (100)	PRJNA630933	124740	Switzerland	FII, FIBpB171		
N1013	*Escherichia coli*	361	NDM-5	pIncF (97)	MW048884	115830	Italy	FII, FIA		
N1014	*Escherichia coli*	1588	NDM-5	pIncF (97)	MW048884	115830	Italy	FII, FIA		
N1070	*Escherichia coli*	405	NDM-5	ND	ND	ND	ND	ND		
N1076	*Escherichia coli*	940	NDM-5	pEsco-5256cz (100)	MG252891	46161	Czechia	X3		
N1081	*Escherichia coli*	361	NDM-5	pIncF (97)	MW048884	115830	Italy	FII, FIA		
N1097	*Escherichia coli*	1431	NDM-5	pEsco-5256cz (100)	MG252891	46161	Czechia	X3		
N1115	*Escherichia coli*	410	NDM-5	pAMA1167-NDM-5 (97)	CP024805	111310	Denmark	FIA, FIB(AP001918), FII(pAMA), Q1		
N1146	*Escherichia coli*	167	NDM-5	pIncF (95)	MW048884	115830	Italy	FII, FIA		
N1153	*Escherichia coli*	167	NDM-5	pIncF (95)	MW048884	115830	Italy	FII, FIA		rmtB
N1235	*Escherichia coli*	1431	NDM-5	pEsco-5256cz (100)	MG252891	46161	Czechia	X3		
N1239	*Escherichia coli*	167	NDM-5	pIncF (97)	MW048884	115830	Italy	FII, FIA		

Isolate	Species	ST	NDM variant	Plasmid mapping/ID (%)	GenBank accession no.	Plasmid size, bp	Country in which described	Replicon	RMTase ID'd in ref plasmid	RMTase ID'd in isolate
N1241	*Escherichia coli*	410	NDM-5	pAMA1167-NDM-5 (97)	CP024805	111310	Denmark	FIA, FIB(AP001918), FII(pAMA), Q1		
N1255	*Escherichia coli*	361	NDM-5	ND	ND	ND	ND	ND		
N1388	*Escherichia coli*	154	NDM-1	ND	ND	ND	ND	ND		
N1416	*Escherichia coli*	405	NDM-5	p2947-NDM-5 (99)	CP046261	66053	China	FII		
N1439	*Escherichia coli*	69	NDM-5	ND	ND	ND	ND	ND		
N1442	*Escherichia coli*	405	NDM-5	ND	ND	ND	ND	ND		
N1452	*Escherichia coli*	167	NDM-5	ND	ND	ND	ND	ND		
N1454	*Escherichia coli*	410	NDM-5	pABC280-NDM-5 (100)	MK372392	35502	UAE	X3		
N1470	*Escherichia coli*	617	NDM-5	pIncF (100)	MW048884	115830	Italy	FII, FIA		
N1494	*Escherichia coli*	410	NDM-5	pNDM-5-IT (96)	MG649062	99476	Italy	FII		rmtB
N1508	*Escherichia coli*	410	NDM-5	pNDM-5-IT (97)	MG649062	99476	Italy	FII		rmtB
N1592	*Escherichia coli*	34	NDM-1	ND	ND	ND	ND	ND		
N1606	*Escherichia coli*	361	NDM-5	p91_NDM-5 (98)	MN007141	125861	Italy	FII, FIA	rmtB	rmtB
N1612	*Escherichia coli*	410	NDM-5	p100_NDM-5 (96)	MN007143	99568	Italy	FII, FIA, FIB(AP001918)		
N1642	*Escherichia coli*	361	NDM-5	pIncF (97)	MW048884	115830	Italy	FII, FIA		
N1644	*Escherichia coli*	361	NDM-5	ND	ND	ND	ND	ND		
N1648	*Escherichia coli*	361	NDM-5	ND	ND	ND	ND	ND		
N1691	*Escherichia coli*	167	NDM-5	pIncF (98)	MW048884	115830	Italy	FII, FIA		
N1700	*Escherichia coli*	69	NDM-1	ND	ND	ND	ND	ND		
N1718	*Escherichia coli*	410	NDM-5	pABC280-NDM-5 (100)	MK372392	35502	UAE	X3		rmtB
N1733	*Escherichia coli*	349	NDM-1	pKP1-NDM-1 (100)	KF992018	137552	Australia	C	rmtC	rmtC
N664	*Klebsiella pneumoniae*	147	NDM-1	pM321-NDM-1 (100)	AP018834	54064	Myanmar	FIB(pQIL)		
N706	*Klebsiella pneumoniae*	147	NDM-1	pM321-NDM-1 (100)	AP018834	54064	Myanmar	FIB(pQIL)		
N735	*Klebsiella pneumoniae*	11	NDM-1	pAR_0146 (100)	CP021962	132217	USA	FII(pKPX1)		
N749	*Klebsiella pneumoniae*	101	NDM-1	ND	ND	ND	ND	ND		rmtC
N768	*Klebsiella pneumoniae*	11	NDM-1	pTR1 (96)	KJ187751	314436	Taiwan	FIIK		
N821	*Klebsiella pneumoniae*	16	NDM-1	pKL8-NDM (100)	MH523448	120614	Italy	FII, FIA, FIB(AP001918)		
N829	*Klebsiella pneumoniae*	147	NDM-1	ND	ND	ND	ND	ND		armA
N900	*Klebsiella pneumoniae*	11	NDM-1	pAR_0146 (100)	CP021962	132217	USA	FII(pKPX1)		
N934	*Klebsiella pneumoniae*	11	NDM-1	pAR_0146 (100)	CP021962	132217	USA	FII(pKPX1)		armA
N940	*Klebsiella pneumoniae*	147	NDM-1	ND	ND	ND	ND	ND		
N972	*Klebsiella pneumoniae*	16	NDM-5	pABC280-NDM-5 (97)	MK372392	35502	UAE	X3		rmtB, rmtF
N1043	*Klebsiella pneumoniae*	4436	NDM-1	pNDM-1-plasmid2 (100)	CP009115	118061	USA	FIIK, FIB(pQIL)		
N1052	*Klebsiella pneumoniae*	395	NDM-1	ND	ND	ND	ND	ND		armA
N1071	*Klebsiella pneumoniae*	147	NDM-1	pM321-NDM-1 (100)	AP018834	54064	Myanmar	FIB(pQIL)		armA
N1075	*Klebsiella pneumoniae*	2735	NDM-5	pEsco-5256cz (100)	MG252891	46161	Czechia	X3		
N1080	*Klebsiella pneumoniae*	11	NDM-1	ND	ND	ND	ND	ND		rmtB
N1086	*Klebsiella pneumoniae*	247	NDM-1	ND	ND	ND	ND	ND		armA
N1095	*Klebsiella pneumoniae*	437	NDM-1	ND	ND	ND	ND	ND		
N1102	*Klebsiella pneumoniae*	101	NDM-4	ND	ND	ND	ND	ND		
N1112	*Klebsiella pneumoniae*	16	NDM-1	pNDM-1-plasmid2 (100)	CP009115	118061	USA	FIIK, FIB(pQIL)		

Isolate	Species	ST	NDM variant	Plasmid mapping/ID (%)	GenBank accession no.	Plasmid size, bp	Country in which described	Replicon	RMTase ID'd in ref plasmid	RMTase ID'd in isolate
N1130	*Klebsiella pneumoniae*	437	NDM-1	pKP1-NDM-1 (95)	KF992018	137552	Australia	C	rmtC	armA
N1154	*Klebsiella pneumoniae*	11	NDM-4	pABC302-NDM-4 (100)	MK372388	49402	UAE	FII, FIA		
N1159	*Klebsiella pneumoniae*	147	NDM-5	pYJ6-NDM5 (99)	AP023236	94613	Myanmar	FII	rmtB	rmtB, rmtF
N1184	*Klebsiella pneumoniae*	11	NDM-5	pEsco-5256cz (100)	MG252891	46161	Czechia	X3		
N1202	*Klebsiella pneumoniae*	437	NDM-1	ND	ND	ND	ND	ND		
N1232	*Klebsiella pneumoniae*	231	NDM-5	pYJ6-NDM5 (98)	AP023236	94613	Myanmar	FII	rmtB	rmtB
N1259	*Klebsiella pneumoniae*	147	NDM-1	ND	ND	ND	ND	ND		armA
N1381	*Klebsiella pneumoniae*	2670	NDM-1	ND	ND	ND	ND	ND		
N1418	*Klebsiella pneumoniae*	147	NDM-5	pYJ6-NDM5 (98)	AP023236	94613	Myanmar	FII	rmtB	rmtB
N1436	*Klebsiella pneumoniae*	147	NDM-1	pM321-NDM-1 (99)	AP018834	54064	Myanmar	FIB(pQIL)		
N1437	*Klebsiella pneumoniae*	392	NDM-1	pYJ6-NDM5 (98)	AP023236	94613	Myanmar	FII	rmtB	
N1448	*Klebsiella pneumoniae*	147	NDM-1	pM321-NDM-1 (100)	AP018834	54064	Myanmar	FIB(pQIL)		armA
N1498	*Klebsiella pneumoniae*	147	NDM-1	ND	ND	ND	ND	ND		armA
N1605	*Klebsiella pneumoniae*	11	NDM-1	pAR_0146 (100)	CP021962	132217	USA	FII(pKPX1)		armA
N1626	*Klebsiella pneumoniae*	11	NDM-1	pAR_0146 (100)	CP021962	132217	USA	FII(pKPX1)		
N1672	*Klebsiella pneumoniae*	147	NDM-1	ND	ND	ND	ND	ND		
N1683	*Klebsiella pneumoniae*	11	NDM-1	pAR_0146 (100)	CP021962	132217	USA	FII(pKPX1)		
N1692	*Klebsiella pneumoniae*	147	NDM-1	pM321-NDM-1 (100)	AP018834	54064	Myanmar	FIB(pQIL)		
N1694	*Klebsiella pneumoniae*	512	NDM-1	pNDM-QD28 (97)	KU167608	46161	China	X3		armA
N1696	*Klebsiella pneumoniae*	395	NDM-1	pKP1-NDM-1 (95)	KF992018	137552	Australia	C	rmtC	armA
N1712	*Klebsiella pneumoniae*	147	NDM-1	pM321-NDM-1 (100)	AP018834	54064	Myanmar	FIB(pQIL)		armA
N1723	*Klebsiella pneumoniae*	76	NDM-1	ND	ND	ND	ND	ND		armA
N727	*Enterobacter cloacae* complex	114	NDM-1	ND	ND	ND	ND	ND		
N840	*Enterobacter cloacae* complex	114	NDM-5	p38_A-OXA140 (95)	CP048377	86996	Switzerland	FIA, FIB(AP001918), FII(pAMA1167-NDM-5)		
N853	*Enterobacter cloacae* complex	136	NDM-1	ND	ND	ND	ND	ND		
N971	*Enterobacter cloacae* complex	121	NDM-1	pNDM-US (95)	CP006661	140825	USA	C	rmtC	armA
N1033	*Enterobacter cloacae* complex	66	NDM-1	pEsco-5256cz (99)	MG252891	46161	Czechia	X3		
N1038	*Enterobacter cloacae* complex	66	NDM-1	pEsco-5256cz (99)	MG252891	46161	Czechia	X3		
N1059	*Enterobacter cloacae* complex	91	NDM-1	pNDM-18ES (100)	MF042350	110432	Romania	FII(Yp)	rmtC	rmtC
N1101	*Enterobacter cloacae* complex	171	NDM-1	pEsST410_NW_NDM (97)	CP031235	85456	UK	M2	armA	armA
N1143	*Enterobacter cloacae* complex	171	NDM-1	pNDM1_045001 (95)	CP043383	85718	China	NT	rmtB	
N1373	*Enterobacter cloacae* complex	231	NDM-1	ND	ND	ND	ND	ND		

Isolate	Species	ST	NDM variant	Plasmid mapping/ID (%)	GenBank accession no.	Plasmid size, bp	Country in which described	Replicon	RMTase ID'd in ref plasmid	RMTase ID'd in isolate
N1405	*Enterobacter cloacae* complex	111	NDM-1	ND	ND	ND	ND	ND		
N1491	*Enterobacter cloacae* complex	78	NDM-1	ND	ND	ND	ND	ND		rmtC
N1497	*Enterobacter cloacae* complex	66	NDM-7	pEsco-5256cz (99)	MG252891	46161	Czechia	X3		
N1730	*Enterobacter cloacae* complex	182	NDM-1	ND	ND	ND	ND	ND		rmtC
N835	*Klebsiella quasipneumoniae*	5330	NDM-1	ND	ND	ND	ND	ND		
N1114	*Klebsiella quasipneumoniae*	4834	NDM-1	ND	ND	ND	ND	ND		
N1598	*Klebsiella quasipneumoniae*	novel	NDM-1	ND	ND	ND	ND	ND		
N676	*Klebsiella aerogenes*	93	NDM-5	pYJ4-NDM-5 (100)	AP023231	83453	Myanmar	FII		
N1431	*Klebsiella aerogenes*	93	NDM-5	pYJ4-NDM5 (97)	AP023231	83453	Myanmar	FII		
N1538	*Klebsiella variicola*	NA	NDM-1	ND	ND	ND	ND	ND		rmtC

ND; not determined; ST, sequence type.
